# Evaluation of Complex Upper Airway Obstruction in Children Using Cine-MRI: A Single-Center Experience and Literature Review

**DOI:** 10.7759/cureus.65025

**Published:** 2024-07-21

**Authors:** Amal R Al-Naimi, Sara G Hamad, Abdusamea Shabani, Mutasim Abu-Hasan

**Affiliations:** 1 Pediatric Pulmonology, Sidra Medicine, Doha, QAT; 2 Pediatric Pulmonary, Hamad Medical Corporation, Doha, QAT; 3 Radiology, Sidra Medicine, Doha, QAT

**Keywords:** cine-mri, obstructive sleep apnea, sleep-related breathing disorder, children, upper airway obstruction

## Abstract

Introduction

Polysomnography (PSG) is considered the gold standard diagnostic test for obstructive sleep apnea (OSA) in children. However, the anatomic location of upper airway obstruction in these patients cannot be determined by PSG, especially in children with complex upper airway obstruction. CT imaging and endoscopic evaluation have been proposed for the evaluation of upper airways in these children. However, cinematic magnetic resonance imaging (Cine-MRI) is a safer, less invasive, and potentially more useful tool for dynamic and anatomical evaluation of upper airways. We here describe the diagnostic outcomes of Cine-MRI in our cohort of children with OSA and suspected complex upper airway obstruction.

Methods

A retrospective chart review of clinical and radiological data of all children with PSG confirmed diagnosis of OSA and who underwent upper airway evaluation using Cine-MRI. Upper airways were evaluated at three different levels: nasopharynx, oropharynx, and supraglottic, during both inspiration and expiration. Fractional collapse (FC) at different levels was used to evaluate dynamic airway collapse and was defined as the difference between maximum and minimum airway dimensions divided by the maximum dimensions.

Results

Eight children (five females and three males) were included. Median age was 8.5 months (range: one month to 16 years). Cine-MRIs identified upper airway obstruction in all patients. Additionally, 50% of the patients had more than one level of obstruction, mainly the nasopharynx and oropharynx. There was a positive correlation between the apnea-hypopnea index (AHI) and FC in the anteroposterior dimension at the nasopharyngeal and the oropharyngeal levels, but it did not reach statistical significance. However, there was a statistically significant negative correlation between AHI and FC in the transverse dimension at the oropharyngeal level. Cine-MRI was helpful in continuous positive airway pressure (CPAP) titration in two patients and was helpful in planning surgical intervention in two patients.

Conclusion

Cine-MRI is a helpful diagnostic tool in evaluating patients with complex upper airway obstruction and can direct potential surgical and non-surgical intervention in pediatric patients with complex upper airway obstruction.

## Introduction

Obstructive sleep apnea (OSA) in children is a very common condition, which is usually caused by tonsillar and/or adenoid hypertrophy. However, on rare occasions, OSA can be caused by complex anatomic narrowing and/or dynamic collapse of the upper airways at one or multiple levels. Diagnosis and treatment of the exact cause of OSA, especially in patients with multiple comorbidities, can be very challenging and require highly specialized diagnostic testing modalities and expertise. Polysomnography (PSG) is considered the gold standard test for the diagnosis of OSA in both children and adults, regardless of the cause. However, PSG is not sufficient for the diagnosis of the nature or the location of the upper airway obstruction [1].

While simple physical examination can detect the common adenoid and tonsillar hyperplasia in children with OSA, more involved methods have been proposed for anatomic and dynamic evaluation of children with complex upper airway obstruction. A CT scan of the upper airways can help in locating sites of anatomic narrowing and the cause of that narrowing, but it is limited in its capacity to detect dynamic collapse of the upper airways as a cause of OSA. On the other hand, drug-induced sleep endoscopy (DISE) is a more helpful method for the detection of dynamic upper airway collapse, but it is limited by the influence of anesthesia on the upper airway dynamics [2]. Cinematic magnetic resonance imaging (Cine-MRI) provides a non-invasive high-resolution anatomic and dynamic examination of the upper airways without the risk of ionizing radiation or need for intervention such as sedation or intubation. It also provides excellent soft tissue contrast and identification of vascular structures [3]. Cine-MRI has been utilized in children with OSA due to complex upper airway obstruction, as well as in children with persistent residual airway obstruction post removal of tonsils and adenoids [4,5].

Cine-MRI is also potentially helpful in planning surgical interventions if needed and in evaluating the effect of applying non-invasive ventilation (NIV) on stenting the upper airways using positive airway pressure and in titrating the required pressure settings [6]. Cine-MRI can also be used to evaluate upper and lower airways in tracheostomized patients [7].

Cine-MRI is a highly specialized technique that requires special expertise and a multidisciplinary approach. Thus far, only a few pediatric centers use Cine-MRI to evaluate patients with complex upper airway obstruction [4,8]. We describe our experience and the clinical and diagnostic outcomes after implementing the Cine-MRI protocol to evaluate patients with complex upper airways.

## Materials and methods

The study is a retrospective review of all children below 18 years of age with PSG-confirmed OSA who had Cine-MRI of the upper airways performed within six months of the PSG. Cine-MRI was done in these children because of the presence of other medical or genetic comorbidities that can potentially cause multilevel anatomic and/or dynamic upper airway obstruction. Tracheostomized children were excluded from the study. The medical electronic records were reviewed and demographic, clinical, and PSG data, as well as Cine-MRI evaluation results, were extracted. Any clinical (i.e., NIV) or surgical interventions based on MRI evaluation were also documented.

All PSG studies were reviewed by the same pulmonologist and scored according to the American Academy of Sleep Medicine (AASM) staging and Scoring Manual V2.5 2018. PSG parameters included the apnea-hypopnea index (AHI), which was calculated as the sum of obstructive apnea and hypopnea events per hour of sleep. The severity of the OSA was categorized according to AHI score: mild if the AHI score was between one and five events/hour, moderate if the AHI score was between 5.1 and 10 events/hour, or severe if the AHI score was above 10 events/hour.

The Cine-MRI was attended, reviewed, and scored by the same radiologist and attended by the treating pulmonologist and respiratory therapist. Upper airways were evaluated for the presence of airway obstruction, and the level of the obstruction. Levels of obstruction considered were the nasopharynx, the oropharynx, and the supraglottic regions. The presence or absence of adenoid or tonsillar hypertrophy was also noted.

Cine-MRIs derived measurements of the upper airways were obtained, including measurement of the maximum and minimum antero-posterior (AP) and transverse diameters at the nasopharyngeal and oropharyngeal levels. Dynamic upper airway obstruction was assessed by measuring airway collapsibility at each level and for each dimension. Fractional collapse (FC) at the nasopharyngeal and oropharyngeal levels was defined as the difference between the maximum (Dmax) and minimum (Dmin) dimensions divided by the maximum dimension (FC = (Dmax - Dmin) / Dmax) [9]. Significant upper airway dynamic collapse was considered present if FC was ≥ 0.5. Glossoptosis was also assessed and was defined as abnormal posterior displacement of the tongue, leading to at least 50% obstruction of the upper airway. Comparison between Cine-MRI-derived indices and PSG parameters was performed using correlation analysis. A P-value of ≤0.05 was considered significant.

Protocol of Cine-MRI

Cine-MRIs are attended by pediatric radiologists, pulmonologists, radiology technicians, and respiratory therapists. Surgical teams (plastic surgery and/or ENT) also attended the Cine-MRI in special cases. The procedure was performed at our institution with the patient breathing spontaneously during natural sleep or while quietly awake. Sedation was not routinely performed. Patients were monitored using pulse oximetry for heart rate and oxygen saturation.

Patients were placed in a supine position with their head and neck fixed straight using a vascular coil. The Ingenia 1.5T MR scanner (Philips, Amsterdam, Netherlands) was used. The MR protocol included the following: T2 fat-saturated sequence in the sagittal plane (FOV 180 mm x 190) 4 x 0.4 mm; T1FFE sequence in the sagittal plane (Cine 50 dynamic runs); T1 FFE in the axial plane at nasopharyngeal and oropharyngeal regions (FOV 250 x 180 mm); and T1 sagittal sequence of the trachea during inspiration and expiration.

Static and dynamic (inspiration vs expiration) upper airway evaluation was done at two separate levels: the nasopharynx and the oropharynx. The nasopharyngeal dimensions were measured at the narrowest point between the posterior aspect of the soft palate and the adenoidal tissue. The oropharyngeal dimensions were measured at the narrowest point between the posterior surface of the tongue and the posterior pharyngeal wall. The maximal dimensions of the airway at each level from the anteroposterior and transverse sections were measured in millimeters.

FC of the nasopharyngeal and oropharyngeal area was calculated by dividing the difference between the maximum and minimum dimensions of the respective area by the maximum dimension [9]. Dynamic collapse was defined as FC of more than 0.80, whereas dynamic motion was defined as FC between 0.50 and 0.80. Stable motion was defined if FC was less than 0.50.

Additionally, adenoidal hypertrophy was identified if the maximal convexity of the adenoidal tissue’s thickness was greater than 12 mm in the posterior nasopharynx on the sagittal Cine-MRIs. Glossoptosis was defined as posterior migration of the tongue, leading to a more than 50% reduction of the airway lumen in the AP dimension.

The study was approved by the Institutional Review Board of Sidra Medicine (IRB#1678170) on 14/1/2021.

## Results

Our study included eight children (five females and three males). Age ranged from one month to 16 years (five patients were less than one year of age). All patients had PSG-confirmed OSA, followed by upper airway evaluation using Cine-MRI. None of the patients required sedation during the Cine-MRI and tolerated the exam well. The median interval between the PSG study and Cine-MRI was 17.5 (1-56) days. PSG studies showed severe OSA in most of the patients (62.5%) with a median AHI of 11 (2.8-28) events per hour. Only one patient was born premature (gestational age: < 37 weeks). Craniofacial anomalies were identified in six (75%) patients. The demographic and clinical data are summarized in Table 1.

**Table 1:** Demographic and clinical data of the participants

Median Age at Cine-MRI in Months (Range)	8.5 (1-199)
	N (%)
Gender	
Male	3 (37.5%)
Female	5 (62.5%)
Prematurity (<37 Weeks Gestational Age)	
Premature	1 (12.5%)
Full term	7 (87.5%)
Nutritional Status	
Failure to thrive	2 (25%)
Normal	6 (75%)
Underlying Disease	
Chromosomal anomalies	3 (37.5%)
Apert syndrome	2 (25%)
Mitochondrial disease	1 (12.5%)
Neurofibromatosis	1 (12.5%)
Mucopolysaccharidose	1 (12.5%)
Craniofacial Anomalies	
Micrognathia	3 (37.5%)
Complex	3 (37.5%)
None	2 (25%)
Severity of OSA	
Mild	2 (25%)
Moderate	1 (12.5%)
Severe	5 (62.5%)
Median apnea-hypopnea index (events per hour) (range) for all the patients	11 (2.8-28)

Upper airway obstruction was identified by Cine-MRI in all patients and revealed different levels of obstruction. The site of the obstruction was identified at the nasopharyngeal level only in one (12.5%) patient, the oropharyngeal level only in one (12.5%) patient, and combined naso-oropharyngeal levels in four (50%) patients, two of these patients had adeno-tonsillar hypertrophy. Glossoptosis was seen in five patients (62.5%), as shown in Table 2.

**Table 2:** Cine-MRI data of the participants

Level of Obstruction Identified on Cine-MRI	N (%)
Single	4 (50%)
Multiple	4 (50%)
Site of Obstruction Identified on Cine-MRI	
Nasopharynx	1 (12.5%)
Oropharynx (retroglossal)	1 (12.5%)
Supraglottic	2 (25%)
Naso- and oro-pharyngeal	4 (50%)
Glossoptosis	
Present	5 (63.5%)
Absent	3 (37.5%)
Nasopharyngeal Airway Measurements	Median (Range)
Fractional collapse (FC, antero-posterior dimension)	0.32 (0.20-0.71)
Fractional collapse (FC, transverse dimension)	0.42 (0.13-0.64)
Retroglossal Airway Measurements	
Fractional collapse (FC, antero-posterior dimension)	0.34 (0.25-0.72)
Fractional Collapse (FC, transverse dimension)	0.28 (0.12-0.57)

**Figure 1:**
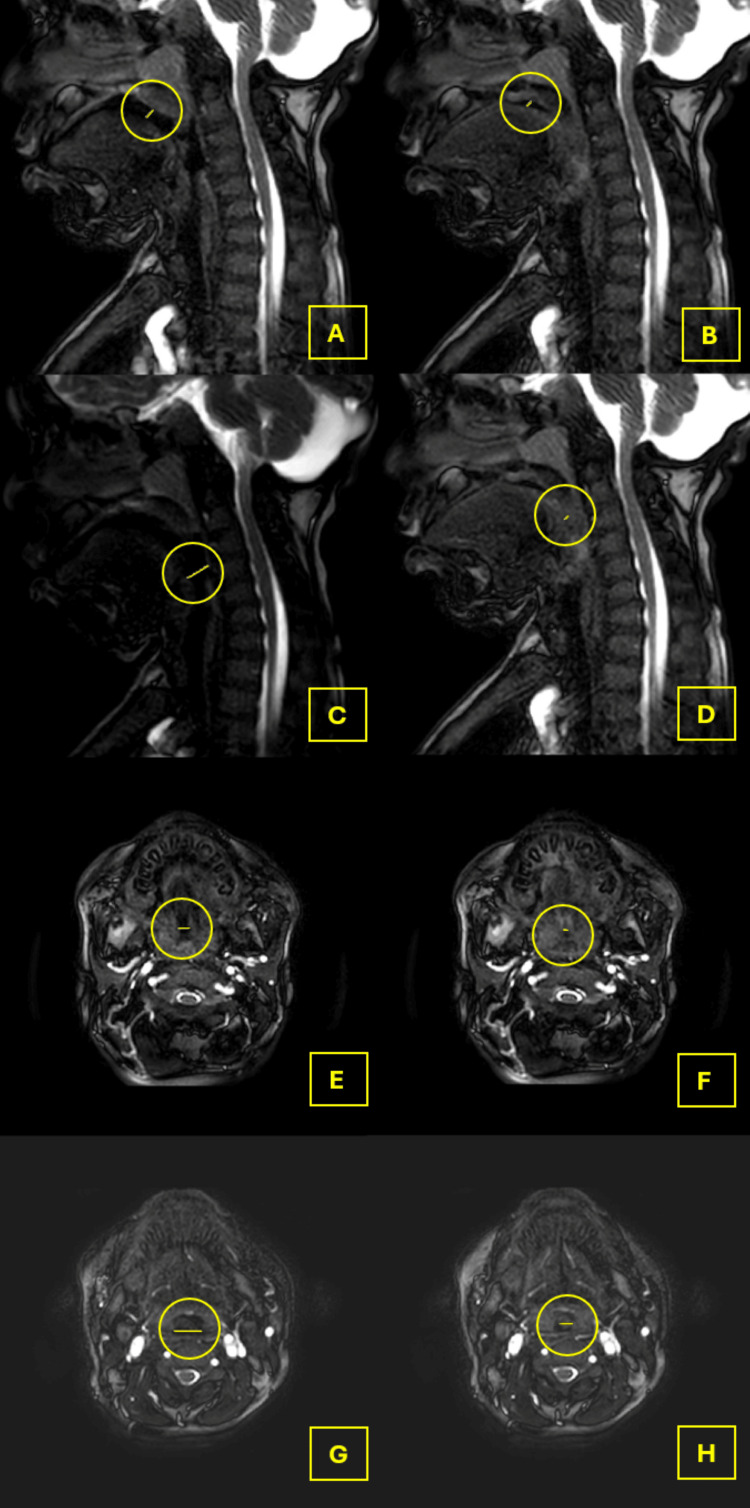
Measurements obtained in Cine-MRI A: A yellow line surrounded by a circle represents the maximal anteroposterior dimension of the nasopharyngeal level. B: A yellow line surrounded by a circle represents the minimal anteroposterior dimension of the nasopharyngeal level. C: A yellow line surrounded by a circle represents the maximal anteroposterior dimension of the oropharyngeal level. D: A yellow line surrounded by a circle represents the minimal anteroposterior dimension of the oropharyngeal level. E: A yellow line surrounded by a circle represents the maximal transverse dimension of the nasopharyngeal level. F: A yellow line surrounded by a circle represents the minimal transverse dimension of the nasopharyngeal level. G: A yellow line surrounded by a circle represents the maximal transverse dimension of the oropharyngeal level. H: A yellow line surrounded by a circle represents the minimal transverse dimension of the oropharyngeal level.

The FC at the nasopharyngeal level in the AP dimension was 0.32 (0.20-0.71), and in the transverse dimension, it was 0.42 (0.13-0.64). Significant upper airway collapse (FC ≥ 0.5) at the level of the nasopharynx was detected in four patients (50%). The measurements of nasopharyngeal airways are shown in AP (maximum: Figure 1A, minimum: Figure 1B) and transverse (maximum: Figure 1E, minimum: Figure 1F) dimensions.

The FC at the oropharyngeal level in the AP dimension was 0.34 (0.25-0.72), and in the transverse dimension, it was 0.28 (0.12-0.57). Significant upper airway collapse (FC ≥ 0.5) was detected in two patients (25%) at the oropharyngeal level. The measurements of oropharyngeal airways are shown in AP (maximum: Figure 1C, minimum: Figure 1D) and transverse (maximum: Figure 1G, minimum: Figure 1H) dimensions.

**Figure 2:**
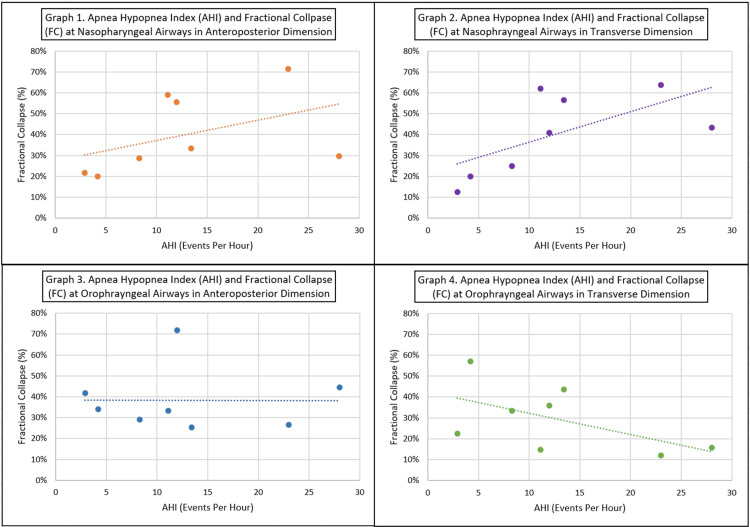
Statistical correlation Graph 1: Correlation graph between the apnea-hypopnea index (AHI) and fractional collapse (FC) at nasopharyngeal airways in anteroposterior dimension. Graph 2: Correlation graph between AHI and FC at nasopharyngeal airways in transverse dimension. Graph 3: Correlation graph between AHI and FC at oropharyngeal airways in anteroposterior dimension. Graph 4: Correlation graph between AHI and FC at oropharyngeal airways in transverse dimension.

No statistically significant correlation was found between AHI and FC at the nasopharyngeal level in either the AP dimensions (P-value of 0.52) or the transverse dimensions (P-value of 0.84). However, a positive trend between AHI and FC of the nasopharynx at both the AP and the transverse dimensions was noted (Figure 2 - Graphs 1 and 2).

There was also no statistically significant correlation between AHI and FC at the oropharyngeal level in the AP dimension (P-value of 0.22). However, there was a statistically significant negative correlation between AHI and FC at the oropharyngeal level in the transverse dimension (P-value of 0.01) (Figure 2 - Graphs 3 and 4).

**Figure 3:**
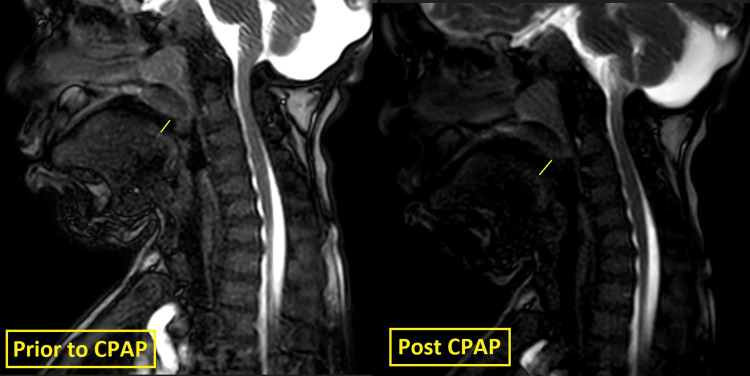
Cine MRI of a patient for titration of continuous positive airway pressure (CPAP) Prior to CPAP, the dimension was 4. Post CPAP, the dimension increased to 6.1. Images are obtained prior and post application of CPAP to show the improvement of minimum anteroposterior dimension (represented by yellow lines) of the oropharynx, which is more than 50%.

During the Cine-MRI examination, titration of the NIV pressures was performed in two patients based on the assessment of the treating team (pulmonologist, respiratory therapist, and radiologist) to achieve at least 50% change in the minimum AP dimension of the affected level of obstruction, as shown in Figure 3.

Moreover, Cine-MRI-guided medical and/or surgical intervention in all patients is based on the identification of the site and the severity of the upper airway obstruction. Two of these patients had midfacial hypoplasia and were planned for craniofacial reconstruction. Two patients had adenotonsillectomy due to hypertrophy causing obstruction at the naso-oropharynx. One patient with midfacial hypoplasia required mandibular distraction, followed by tracheostomy tube insertion due to persistent upper airway obstruction. One patient had hypotonia with severe laryngomalacia and required a tracheostomy tube. All patients improved clinically after the intervention. Patients’ characteristics, Cine-MRI findings, and performed interventions are summarized in Table 3.

**Table 3:** Patients’ characteristics, Cine-MRI findings, and performed interventions

Patient	Age	Diagnosis	Obstructive sleep apnea severity	Multiple levels of obstruction	Glossoptosis	Site of airway obstruction per Cine-MRI	Intervention (during Cine-MRI)	Surgical Intervention
1	3 months	Chromosome 9 deletion	Severe	No	Yes	Oropharyngeal	None	Mandibular distraction followed by tracheostomy
2	10 months	Neurofibromatosis adenotonsillar hypertrophy	Severe	Yes	No	Naso- and oropharyngeal	None	Adenotonsillectomy
3	1 month	Apert syndrome	Mild	Yes	Yes	Naso- and oropharyngeal	None	Craniofacial reconstruction
4	2 years	Chromosome 5 deletion adenotonsillar hypertrophy	Severe	Yes	No	Naso- and oropharyngeal	None	Adenotonsillectomy
5	16 years	Mucopolysaccharidosis	Severe	Yes	Yes	Naso- and oropharyngeal	CPAP titration	None
6	6 years	Apert syndrome	Mild	No	Yes	Nasopharyngeal	None	Craniofacial reconstruction
7	7 months	Chromosome 5 deletion micrognathia severe laryngomalacia	Moderate	No	Yes	Supraglottic	CPAP titration	Tracheostomy
8	6 months	Mitochondrial disease	Severe	No	Yes	Supraglottic	CPAP titration	None

## Discussion

Cine-MRI is becoming a valuable tool for airway evaluation, particularly in patients with complex airway obstruction who may require further surgical interventions. Therefore, we were interested in establishing a Cine-MRI protocol in our hospital to standardize the procedure and help in planning further surgical and non-surgical interventions, including NIV support. In this study, we reviewed the diagnostic and therapeutic outcomes of our Cine-MRI protocol. We showed that Cine-MRI helped in identifying the anatomical location of the airway obstruction in our patients and guided further surgical intervention, as well as NIV titration. All our patients did not require sedation during the Cine-MRI, which was intentionally avoided to decrease its effect on the upper airway muscle tone [10]. All patients tolerated the procedure well. The presence of the treating pulmonologist and respiratory therapist helped in NIV management during the procedure, which provided an added therapeutic value.

The majority of our patients had combined nasopharyngeal and oro-pharyngeal obstruction based on the radiologist's assessment. To objectively evaluate the airway obstruction at both levels, we applied a proposed method in the literature calculating the airway caliber and airway FC ratio. A decrease in the airway caliber during the respiratory cycle by 50% or more has been considered abnormal [8,9]. In our study, five out of the eight patients showed significant airway collapsibility (FC ≥ 5). However, there was no significant correlation between AHI and FC even at the nasopharynx level despite the positive trend between both.

Few previous studies had evaluated the diagnostic value of Cine-MRI in children with OSA using variable outcomes. Cappabianca et al. retrospectively assessed 80 children (age: 4-15 years), 40 children with OSA, and 40 children without OSA. The aim of the study was to examine the anatomical areas of airway obstruction in children with OSA other than adenoids and tonsils. The OSA group had significantly smaller nasopharyngeal and oropharyngeal cross-section areas. The soft palate volume, adenoid, and tonsils were significantly larger in children with OSA. Additionally, the OSA group had a smaller mandibular volume and a lower position of the hyoid bone [11]. Arens et al. used Cine-MRI to define the upper airway in 20 children with OSA in comparison to 20 controls (age: 1.9-7.9 years versus 1.9-7.8 years, respectively). They showed that the upper airway's mean cross-sectional area, minimal cross-sectional area, and its volume in the OSA group was significantly smaller in comparison to the control group (p < 0.0005). They concluded that the upper airway in children with OSA is narrower along its initial length [12]. Zeng et al. used Cine-MRI to evaluate 51 children with OSA (3-13 years of age) with a mean AHI of 20.0  ± 8.0. They showed a positive correlation between AHI and the ratio of adenoid area to nasopalatine pharyngeal cavity (Sa/Snp) and a negative correlation between the Sa/Snp and the lowest oxygen saturation during sleep [13]. The previous studies confirmed the role of adenotonsillar hypertrophy as a cause of OSA but also showed other potential causes of upper airway obstruction in patients with OSA.

On the other hand, some studies focused on children with Down syndrome and children with persistent OSA post adenotonsillectomy. Donnelly et al. studied 27 patients with Down syndrome (age range: 4-19 years) with persistent OSA post adenotonsillectomy and found that the majority of patients had airway obstruction at multiple levels, including macroglossia in 20 patients (74%), glossoptosis in 17 (63%), recurrent and enlarged adenoid tonsils in 17 (63%), enlarged lingual tonsils in eight (30%), and hypopharyngeal collapse in six (22%) [14]. Isaiah et al. evaluated 36 patients with persistent OSA post adenotonsillectomy (aged 3-18 years), mostly with Down syndrome (47%), and 17% were obese. The airway size estimation was based on measuring the maximum area (mean ± SD), cm^2^; minimum area (mean ± SD) cm^2^; and relative narrowing (max-min/max area, mean ± SD) cm^2^. A single site of obstruction was identified in 58% of the patients, and 33% had a multilevel obstruction. Multiple regression analysis showed that a combination of the minimum airway diameter and body mass index z-score predicted OSA severity [15]. Clark et al. [16] retrospectively compared the results of Cine-MRI and drug-induced sleep endoscopy (DISE) in detecting airway obstruction in 15 children with persistent OSA after adenotonsillectomy (age: 7-18 years), 60% were Down syndrome, and 53% of the patients were obese. The AHI ranged from 6.2 to 98.6 (moderate OSA in five patients and severe in 10 patients). DISE and Cine-MRI revealed a single site of obstruction in the majority of patients (73%) at the level of the tongue base [16]. Nandalike et al. studied 27 obese children with OSA (age: 8-17 years, body mass index Z-score: 2.5 ± 0.3) who underwent polysomnography and head MRI before and after adenotonsillectomy. Only 12 patients (44%) had normal AHI post adenotonsillectomy (AHI dropped from 23.7 ± 21.4 to 5.6 ± 8.7). Improvement in the AHI was less in the patients with severe OSA. They related that to the significant residual adenoid tissue and increase in the volume of the tongue and soft palate [17]. The above studies and other studies mentioned in Table 4 showed that Cine-MRI can help in exploring the causes of persistent OSA post adenotonsillectomy, especially in Down syndrome and obese patients [8-21]. Cine-MRI can explain the low success rate of adenotonsillectomy in high-risk patients, which can help prospectively in choosing the appropriate surgical approach. Cine-MRI was also employed as a method to assess the upper airway's response to changes in pressure, as previously proposed [22].

**Table 4:** Summary of published studies about Cine-MRI

Study, Year Published	Population (N)	Age	Apnea Hypopnea Index (AHI)	Intervention	Cine-MRI Results in Obstructive Sleep Apnea (OSA) Group
Cappabianca et al., 2013 [11]	OSA (40) vs control (40)	4-15 years	OSA group, mean ± SD (8.1±3.5)	None	Small naso-oropharyngeal cross-section area. Adenotonsillar hypertrophy. Small mandibular volume & lower position of the hyoid bone
Arens et al., 2003 [12]	OSA (20) vs control (20)	1.9-7.9 years	OSA group, mean ± SD (8.4±9.5)	None	Upper airway in the OSA group was narrow along the initial 60–70% predominantly where the adenoid and tonsils overlap
Zeng et al., 2018 [13]	OSA (52)	3-13 years	Mean ± SD (20.0 ± 8.0)	None	Adenoidal hypertrophy
Donnelly et al., 2004 [14]	Trisomy 21 with OSA (27)	4-19 years	No	Post adenotonsillectomy	Glossoptosis (63%). Recurrent & enlarged adenoid tonsils (63%). Enlarged lingual tonsils (30%)
Shott et al., 2004 [18]	Trisomy 21 with OSA (15)	1-14 years	Range (1.9-15)	Post adenotonsillectomy	Glossoptosis (80%). Nasopharyngeal collapse (60%). Adenoid regrowth (53%)
Isaiah et al., 2018 [15]	OSA (36) (47% Trisomy 21)	3-18 years	Mean ± SD (15.5±18.1)	Post adenotonsillectomy	Obstruction at the tongue base (89%). Obstruction at the Nasopharynx (36%)
Clark et al., 2017 [16]	OSA (15) (60% Trisomy 21)	7-18 years	Range (6.2-98.6)	Post adenotonsillectomy	Obstruction at the tongue base
Nandalike et al., 2013 [17]	Obese with OSA (27)	8-17 years	Mean ± SD (5.6±8.7)	Post adenotonsillectomy	Residual adenoid tissue. Increase in the volume of the tongue and soft palate
Prosser et al., 2017 [19]	Trisomy 21 (21)	4.4-17.2 years	Range (3.8-43.8)	Post adenotonsillectomy	Enlarged lingual tonsils
Propst et al., 2017 [20]	Trisomy 21 (13) 54% Obese	6.8-18.7 years	Range (14.5-114.2)	Post adenotonsillectomy	Relative macroglossia (100%). Lingual tonsil hypertrophy (92%). Glossoptosis (85%)
Wootten et al., 2010 [21]	OSA (31) (61% Trisomy 21)	3.1-23 years	Mean ± SD (14.1±10.1)	Post adenotonsillectomy	Tongue base obstruction

Limitation of the study

Limitation of the study includes its retrospective design, small sample size, and variability in pathologies. However, our study had more complex patients and younger age groups, including infants than previously reported articles and highlighted the therapeutic implication of Cine-MRI, which aided in the decision-making of these patients and resulted in improved outcomes. While some patients required further surgical interventions due to the multilevel airway obstruction, other patients had their NIV settings titrated using Cine-MRI.

## Conclusions

In conclusion, Cine-MRI is an attractive diagnostic tool for evaluating pediatric patients with upper airway obstruction, especially in the perioperative assessment of patients with complex airway obstruction. Future studies are crucial to standardizing Cine-MRI, methodology, and terminology. Reliable airway measurements that capture both fixed and dynamic airway obstruction in an objective way are also needed. Finally, normal reference values for upper airway size during the respiratory cycle in children are necessary to define fixed versus dynamic upper airway obstruction during Cine-MRI.
